# FOXF2 deficiency promotes epithelial-mesenchymal transition and metastasis of basal-like breast cancer

**DOI:** 10.1186/s13058-015-0531-1

**Published:** 2015-02-26

**Authors:** Qing-Shan Wang, Peng-Zhou Kong, Xiao-Qing Li, Fan Yang, Yu-Mei Feng

**Affiliations:** Department of Biochemistry and Molecular Biology, Tianjin Medical University Cancer Institute and Hospital, National Clinical Research Center of Cancer, Huan-Hu-Xi Road, Tianjin, 300060 China; Key Laboratory of Breast Cancer Prevention and Treatment of the Ministry of Education, Tianjin Medical University Cancer Institute and Hospital, National Clinical Research Center of Cancer, Huan-Hu-Xi Road, Tianjin, 300060 China

## Abstract

**Introduction:**

Our previous clinical study demonstrated that the under-expression of *FOXF2* is associated with early-onset metastasis and poor prognosis of patients with triple-negative breast cancer. In this study, we further characterized the role of FOXF2 in metastasis of basal-like breast cancer (BLBC) and underlying molecular mechanisms.

**Methods:**

RT-qPCR, immunoblot, immunofluorescence and immunohistochemistry were performed to assess the expression of genes and proteins in cell lines and tissues. A series of *in vitro* and *in vivo* assays was performed in the cells with RNAi-mediated knockdown or overexpression to elucidate the function and transcriptional regulatory role of FOXF2 in breast cancer.

**Results:**

We found that FOXF2 was specifically expressed in most basal-like breast cells. FOXF2 deficiency enhanced the metastatic ability of BLBC cells *in vitro* and *in vivo.* Additionally, FOXF2 deficiency induced the epithelial-mesenchymal transition (EMT) of basal-like breast cells. Furthermore, we identified that *TWIST1* is a transcriptional target of FOXF2. TWIST1 was negatively regulated by FOXF2 and mediated the FOXF2-regulated EMT phenotype of basal-like breast cells and aggressive property of BLBC.

**Conclusions:**

FOXF2 is a novel EMT-suppressing transcription factor in BLBC. FOXF2 deficiency enhances metastatic ability of BLBC cells by activating the EMT program through upregulating the transcription of *TWIST1*.

**Electronic supplementary material:**

The online version of this article (doi:10.1186/s13058-015-0531-1) contains supplementary material, which is available to authorized users.

## Introduction

Breast cancer is the most frequently diagnosed cancer among females [1]. Metastasis is the leading cause of death in breast cancer patients. To develop distant metastatic disease, the epithelial cancer cells need to undergo a morphological change into a mesenchymal phenotype to acquire migration and invasion ability [2]. Epithelial-mesenchymal transition (EMT) is a program of switching polarized epithelial cells to migratory mesenchymal cells [2]. The aberrant activation of the EMT program is implicated in tumor progression, metastasis and acquisition of therapeutic resistance [2,3]. EMT-transcription factors (EMT-TFs) such as TWIST1, SNAIL1, SNAIL2, ZEB1, ZEB2, Goosecoid and FOXC2, can trigger the EMT program by directly or indirectly suppressing E-cadherin expression [4-7]. TWIST1 is a member of the basic helix-loop-helix (bHLH) transcription factor family [8] and plays a pivotal role in the regulation of embryogenesis, gastrulation and mesoderm formation during early embryonic development [9,10]. *TWIST1* was found to be frequently activated in a wide array of human cancers and is associated with poor prognosis [3,11]. TWIST1 induces the EMT program by downregulating E-cadherin expression through indirect effects on the *CDH1* promoter [3].

Breast cancer is a heterogeneous disease. Based on their gene expression profiles, breast cancers can be classified into distinct molecular subtypes: normal breast-like, luminal A, luminal B, human epidermal growth factor receptor 2 (HER2)-enriched, and basal-like [12]. Basal-like breast cancer (BLBC) is less likely to express estrogen receptor (ER), progesterone receptor (PR) and HER2, which are also characteristics of triple-negative breast cancer (TNBC). Thus, BLBC shares many features with TNBC, and the two terms are often used interchangeably [13]. In addition to the triple-negative marker status, BLBC is characterized by the expression of basal markers such as cytokeratins (CK) 5/6, CK14, CK17 and epithelial growth factor receptor (EGFR) in the clinic [12]. TNBC and/or BLBC is recognized as a particularly aggressive subtype and receives less benefit from targeted therapy [12]. Therefore, there is an urgent need to elucidate the molecular pathogenesis of TNBC and/or BLBC and develop effective systemic therapies, especially molecular-targeted therapy.

Recent reports have revealed that TNBC/BLBC is a group of heterogeneous tumors [14]. BLBC also can be divided into extraordinarily diverse basal-like A and basal-like B subtypes [15]. The basal-like A cells have either luminal-like or basal-like epithelial morphology, while the basal-like B cells appear poorly differentiated and possess more mesenchymal characteristics [15]. Thus, the basal-like B subtype is more aggressive than the basal-like A subtype [15]. Due to the heterogeneity of BLBC, it is important to identify the critical regulatory factors that are associated with aggressive phenotype of BLBC. It is well known that various embryonic and mesenchymal EMT-TFs, including SNAIL1 [16], SNAIL2 [17] TWIST1 [18], and Forkhead box (FOX) transcription factor superfamily members FOXC1 [19], FOXC2 [4] and FOXQ1 [20], contribute to the aggressive phenotype of BLBC.

The mesenchymal regulator FOXF2 belongs to the FOX transcription factor superfamily [21]. It is specifically expressed in the mesenchyme adjacent to the epithelium in organs derived from the splanchnic mesoderm [22], and plays an important role in tissue homeostasis through regulating epithelium-mesenchyme interaction to maintain epithelium polarity [22]. Our previous clinical study demonstrated that the under-expression of *FOXF2* is associated with early-onset metastasis and poor prognosis of patients with TNBC, but not the prognosis of non-TNBC patients [23]. This result suggests that FOXF2 deficiency is involved in TNBC/BLBC metastasis through regulating EMT. Recent studies have indicated that FOXF2 is a potential tumor suppressor in both prostate cancer [24] and breast cancer [25]. However, the role of FOXF2 in breast cancer metastasis and the underlying molecular mechanisms remain largely unknown.

In this study, we identified FOXF2 as a novel EMT-suppressing transcription factor in BLBC and demonstrated that it directly represses the transcription of *TWIST1*. FOXF2 deficiency promotes metastasis of BLBC cells through transcriptional upregulation of *TWIST1* and activation of EMT.

## Materials and methods

### Cell culture

The human breast cancer cell lines MDA-MB-231, BT549, MCF-7, BT474, ZR-75-30, SKBR-3 and MDA-MB-453, immortalized non-tumorigenic basal-like mammary epithelial cell lines MCF-10A and HBL100 were obtained from American Type Culture Collection (Manassas, VA, USA). MDA-MB-231-luc-D3H2LN (231-Luc), a MDA-MB-231 subclone expressing luciferase, was obtained from Caliper Life Sciences (Hopkinton, MA, USA). MCF-10A cells were maintained in DMEM-F12 medium (Invitrogen, Gaithersburg, MD, USA) supplemented with 5% horse serum, 20 ng/mL EGF, 500 ng/mL hydrocortisone, 10 μg/mL insulin and 100 ng/mL cholera toxin. The other cells were cultured in DMEM-F12 (MCF-7 and MDA-MB-453) or RPMI 1640 (MDA-MB-231, BT549, BT474, ZR-75-30, SKBR-3 and HBL100) medium (Invitrogen) supplemented with 10% fetal bovine serum (FBS; Invitrogen), 100 units/mL penicillin, and 100 mg/mL streptomycin (Invitrogen). All cell lines were incubated in a humidified incubator at 37°C with 5% CO_2_ and grown into logarithmic phase and/or 80% confluence for the experiments.

### Lentiviral transduction of shRNA and transfection of interfering RNA and plasmids

To obtain the stable *FOXF2*-knockdown cells, cells were infected with the lentiviral vector expressing a short hairpin RNA (shRNA) targeting the sequence GTCCTCAACTTCAATGGGATT in the coding sequence region of human *FOXF2* gene (shFOXF2; Sigma-Aldrich, St Louis, MO, USA; TRCN0000013959). A shRNA non-targeting human and mouse gene was used as control (shControl; Sigma-Aldrich; SHC002). The cells were selected in 2 μg/mL puromycin to establish stable expressing shRNAs cells. For the rescue experiment, the stable *FOXF2*-knockdown cells were transfected with shRNA-resistant full-length *FOXF2* plasmid (Genechem, Nanjin, China), which has three nucleotide mismatches within the target sequence of the shFOXF2 (GTCCTCAATTTTAACGGGATT). To silence the expression of *TWIST1*, the cells were transiently transfected with two independent small interfering RNAs (siRNAs) targeting the coding sequence region of human *TWIST1* gene GGATCAAACTGGCCTGCAA (si-TW#1) and GAACACCTTTAGAAATAAA (si-TW#2; RiboBio Co., Guangzhou, China) or a negative control siRNA (si-Control) using Lipofectamine 2000 (Invitrogen) according to the manufacturer’s specifications. The human full-length *FOXF2* cDNA was amplified from a pEV-FOXF2 plasmid, which was a gift from Professor Peter Carlsson (University of Gothenburg, Department of Cell and Molecular Biology, Sweden), and then was subcloned into the pcDNA3.1-HA vector (Invitrogen). The human full-length *TWIST1* cDNA (Genechem) was subcloned into the pcDNA3.1 vector (Invitrogen). The cells were transfected with pcDNA3.1-HA-FOXF2 (FOXF2), pcDNA3.1-TWIST1 (TWIST1) or vector control (Invitrogen) using Lipofectamine 2000.

### Reverse transcription quantitative polymerase chain reaction

Total RNA isolation from cultured cells or tissues, reverse transcription (RT) reactions, quantitative PCR (qPCR), and the quantification of target gene expression were performed as previously described [23].

### Immunoblot, immunohistochemistry, and immunofluorescence

The protocols for protein analysis by immunoblot, immunohistochemistry, and immunofluorescence were performed as previously described [26]. The primary antibodies were anti-E-cadherin, anti-N-cadherin, anti-vimentin (BD Biosciences, Bedford, MA, USA), anti-FOXF2 (Abnova, Aachen, Germany), anti-TWIST1, anti-HA tag (Abcam, Cambridge, MA, USA), and anti-β-actin (Sigma-Aldrich).

### Chromatin immunoprecipitation assay

Chromatin immunoprecipitation (ChIP) assay was performed using a ChIP assay kit (EMD Millipore, Billerica, MA, USA) according to the manufacturer’s instructions. Anti-HA antibody (Abcam) was used for immunoprecipitation to enrich the promoter fragments containing putative FOXF2 binding site of target genes in MCF-10A cells constitutively expressing HA-tagged FOXF2 (MCF-10A-FOXF2). The isotype IgG (Abcam) was used as a negative control. The primers for the amplification of the *TWIST1* promoter region containing a putative FOXF2 binding site from −2120 to −2113 relative to the transcription start site (TSS) were 5′-AGATTTCCTTTACACTTTACCC-3′ (forward) and 5′-GCGAGTGTTATTTCTCCAGCGA-3′ (reverse). The primers for the amplification of *TWIST1* exon 1 (+408 to +841), which served as a negative control, were 5′-CAGCGAGGAAGAGCCAGACCG-3′ (forward) and 5′-GGAGGACCTGGTAGAGGAAGT-3′ (reverse).

### Luciferase reporter assay

The *TWIST1* promoter region with (−2140 to +27) or without (−2057 to +27) FOXF2 binding sites was amplified from human genomic DNA using the primers 5′-AGTCCAATCATTCGATCTC-3′ (forward) and 5′-CTGCAGACTTGGAGGCT-3′ (reverse), or 5′-ATAGCTGAAGTGGAAAAGG-3′ (forward) and 5′-CTGCAGACTTGGAGGCT-3′ (reverse) with Kpn I and Bgl II restriction endonuclease recognition sites at the 5′-ends, respectively. The fragments of the *TWIST1* promoter were cloned into the luciferase reporter gene plasmid pGL3-Basic (Promega, Madison, WI, USA). The pGL3 reporter and pRL-TK plasmid were transiently co-transfected into MCF-10A and BT549 cells for 48 h. The luciferase activity of pGL3-TWIST1 (−2140/+27) or pGL3-TWIST1 (−2057/+27) was normalized to Renilla luciferase activity.

### Cell proliferation

Cell proliferation ability was assessed using a 3-(4,5-dimethylthiazol-2-yl)-2,5-diphenyltetrazolium bromide (MTT) assay. Cells were seeded at a density of 2 × 10^3^ cells/well in 96-well plates. On day 1, 2, 3, 4 and 5, the cells were incubated with 10 μL MTT solution (5 mg/mL in phosphate-buffered saline (PBS)) at 37°C for 4 h. After removal of the medium, 100 μL DMSO was added to each well and absorbance was measured at 570 nm.

### Cell invasion and migration assay

Cell invasion and migration ability *in vitro* were assessed using Matrigel-coated and non-Matrigel-coated transwell inserts (BD Biosciences) as described previously [26].

### Xenograft tumor assay

Twenty female 4- to 6-week-old severe combined immunodeficiency (SCID) mice were randomly divided into two groups for the xenograft tumor assay. Then, 5 × 10^6^ 231-Luc cells infected with shFOXF2 (231-Luc-shFOXF2) or shControl (231-Luc-shControl) were washed and harvested in 0.1 mL PBS and injected into the left lower abdominal mammary fat pad of the SCID mice [27]. After 22 days, all mice were anesthetized using isoflurance and injected intraperitoneally with 150 mg/kg D-luciferin/PBS. The bioluminescent imaging was performed using a Xenogen IVIS system (Caliper Life Sciences, Hopkinton, MA, USA). The data of living image were expressed as photon flux (photons per second). The tumor volumes were measured by caliper at the indicated time and calculated using the formula (length x width^2^)/2 [28]. The survival time of mice was defined as the time interval between the day of cell injection and the time of death or sacrifice. The xenograft tumors, livers and lungs were harvested from xenograft mice at sacrifice. The tissues were prepared for paraffin-embedded tissue section and hematoxylin and eosin (H&E) staining or immunohistochemistry staining. Two independent experiments were performed with xenograft mice. In one experiment, the mice died naturally to allow survival time calculations. In the second experiment, the mice were sacrificed for dissection on day 21 after cell injection. The mouse xenograft tumor assays were performed in accordance with protocols approved by the Animal Ethics Committee of Tianjin Medical University Cancer Institute and Hospital (TMUCIH; Tianjin, China. No. 058).

### Breast cancer tissue specimens

A total of 34 primary TNBC tissue specimens were obtained from patients who underwent breast surgery in TMUCIH. The use of these specimens was approved by the Institutional Review Board of TMUCIH, and written consent was obtained from all participants. All cases were followed up over 3 years, and 30 cases were followed up over 5 years. Disease-free survival (DFS) was defined as the time interval between primary surgery and any relapse (local-regional, contra-lateral and/or distant), or the terminal time of follow-up without any relapse events.

### Independent data sets for validation of gene expression levels in breast cancer cell lines and breast cancer tissues

The *FOXF2* mRNA expression pattern in different subtypes of breast cell lines was validated using the Gene expression-based Outcome for Breast cancer Online (GOBO) data sets [29].

### Statistical analysis

Data from *in vitro* and *in vivo* experiments were presented as mean ± standard deviation (SD). Student’s *t* test was used to compare differences between the experimental group and the control group. Fisher’s exact test was used to compare the difference of metastatic incidence between 231-Luc-shFOXF2 mice and the 231-Luc-shControl mice. Spearman’s rank correlation was used to analyze the correlation between *FOXF2* and *TWIST1* mRNA levels in breast cancer tissues. Survival plots were created using Kaplan-Meier analysis, and log-rank test was used to assess statistical significance. *P* <0.05 was considered statistically significant.

## Results

### FOXF2 deficiency enhances migration and invasion of basal-like mammary epithelial cells and BLBC cells *in vitro*

**Figure 1:**
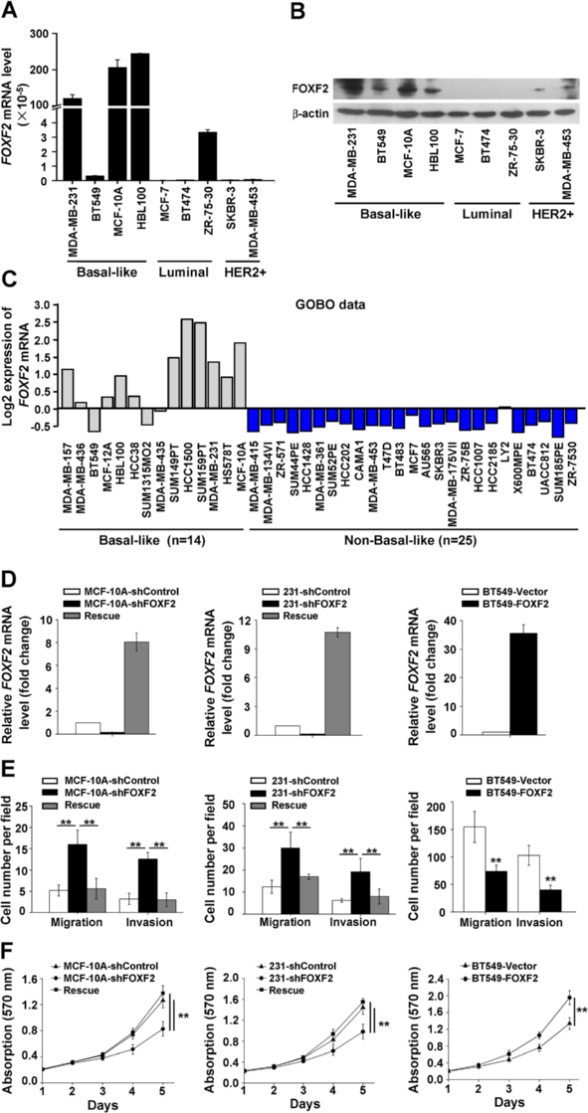
**FOXF2 deficiency enhances migration and invasion of basal-like mammary epithelial cells and breast cancer cells** ***in vitro.*** **(A)** *FOXF2* mRNA levels in the indicated cell lines were measured by RT-qPCR. The data were normalized to the expression of the housekeeping gene glyceraldehyde 3-phosphate dehydrogenase (*GAPDH*). **(B)** The protein expression of FOXF2 in the indicated cells was detected by immunoblot. **(C)** *FOXF2* mRNA levels in different subtypes of breast cell lines were analyzed using an independent GOBO dataset. **(D)** *FOXF2* mRNA levels in the indicated cells were measured by RT-qPCR. Fold changes were relative to the mRNA expression of the control cells. **(E)** The migration and invasion ability of the indicated cells were assessed by transwell assay. **(F)** Proliferation of the indicated cells was determined by MTT assay. Three independent assays were performed in triplicate. The data were expressed as mean ± SD. ^**^ *P* <0.01. GOBO, Gene expression-based Outcome for Breast cancer Online; MTT, 3-(4,5-dimethylthiazol-2-yl)-2,5-diphenyltetrazolium bromide; RT-qPCR, reverse transcription quantitative PCR; SD, standard deviation.

FOXF2 mRNA and protein expression levels in various breast cell lines, including basal-like subtype cells MDA-MB-231, BT-549, MCF-10A, HBL100 and non-basal-like cells MCF-7, BT474, ZR-75-30, SKBR-3 and MDA-MB-453, were measured by RT-qPCR and immunoblot, respectively. The results showed that FOXF2 was highly expressed in most basal-like cell lines and was less expressed in non-basal-like cell lines (Figure 1A and B). This expression pattern of *FOXF2* in the basal-like (n = 14) and non-basal-like (luminal; n = 25) subtype breast cell lines was validated using GOBO data set analysis (Figure 1C). The result confirmed that FOXF2 was frequently and highly expressed in mesenchymal/myoepithelium-like breast cells. Combining these results with our previous finding that the under-expression of *FOXF2* is associated with early-onset metastasis and poor prognosis of TNBC patients, but not the prognosis of non-TNBC patients [23], we hypothesized that FOXF2 may play a role in the metastasis of BLBC.

**Figure 2:**
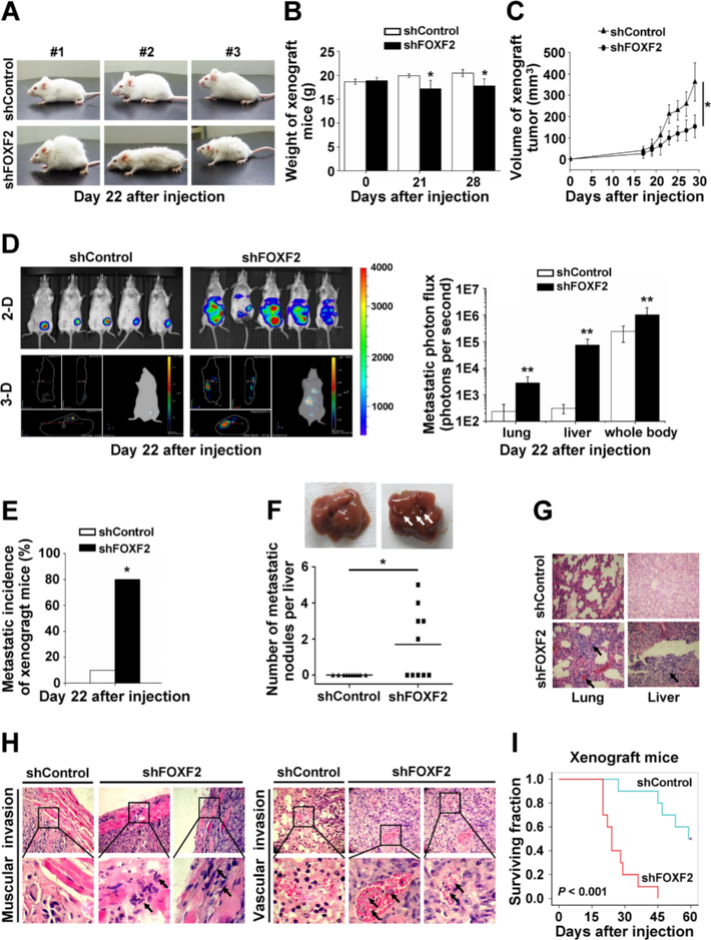
**FOXF2 deficiency enhances metastatic ability of BLBC cells** ***in vivo.*** **(A)** Representative photos of the general physical condition of xenograft mice injected with MDA-MB-231-Luc-shFOXF2 or the control cells on day 22 after injection. **(B)** The weights of xenograft mice injected with MDA-MB-231-Luc-shFOXF2 (n = 4-10) or the control cells (n = 9-10) were measured at the indicated times. **(C)** Tumor volume of xenograft mice injected with MDA-MB-231-Luc-shFOXF2 (n = 3-10) or the control cells (n = 9-10) at the indicated times. **(D)** Bioluminescence images of primary xenograft tumor and metastatic tumor of xenograft mice injected with MDA-MB-231-Luc-shFOXF2 (n = 7) or the control cells (n = 10) on day 22 after injection (Left) and the quantitation of metastatic photon flux of the lung, liver and whole body (Right). **(E)** Metastatic incidence of xenograft mice injected with MDA-MB-231-Luc-shFOXF2 cells (n = 10) and the control cells (n = 10) on day 22 after injection. **(F)** Representative photos (Top) and count (Bottom) of visible metastatic nodules in the liver of euthanized xenograft mice injected with MDA-MB-231-Luc-shFOXF2 cells (n = 10) and the control cells (n = 10) on day 21 after injection. **(G)** Representative H&E stained sections of lung and liver harvested from xenograft mice. The arrows point to the metastatic foci in lung and liver. **(H)** Representative H&E-stained sections of primary tumor harvested from xenograft mice. The arrows indicate that the cancer cells invaded into muscle (Left) and vasculature (Right). The selected areas of muscular and vascular invasion are enlarged in the respective bottom panels. **(I)** Kaplan-Meier curves of the overall survival of xenograft mice (n = 10). Two independent experiments were performed. The data were expressed as mean ± SD. ^*^ *P* <0.05; ^**^ *P* <0.01. BLBC, basal-like breast cancer; H&E, hematoxylin and eosin; SD, standard deviation.

To investigate the roles of FOXF2 in regulating the invasive potential of BLBC cells *in vitro*, the immortalized non-tumorigenic basal-like mammary epithelial cell line MCF-10A and the invasive BLBC cell lines MDA-MB-231 was selected to establish stable *FOXF2*-knockdown cells by infection with a lentiviral vector expressing a *FOXF2* shRNA (shFOXF2) or a non-target shRNA control lentiviral vector (shControl). The knockdown efficiency of FOXF2 in these cells was shown in Figures 1D and 2B. Boyden chamber transwell assays showed that *FOXF2* knockdown significantly promotes migration and invasion of both the two cell lines (Figure 1E). To exclude the off-target effects of the shFOXF2, we performed a rescue experiment by transfecting a shRNA-resistant full-length *FOXF2* in the stable *FOXF2*-knockdown cells. The results showed that the effect of shFOXF2 on migration and invasion of MCF-10A and MDA-MB-231 cells was reversed (Figure 1E). Next, we transiently transfected pcDNA3.1-FOXF2 in the BT549 cells, a BLBC cell line with high invasiveness and less FOXF2 expression. The overexpressed FOXF2 in the cells was shown in Figures 1D and 2B. We observed that FOXF2 overexpression significantly decreased the migration and invasion of BT549 cells (Figure 1E). In contrast with the role of promoting cell migration and invasion, the cell growth curves showed that FOXF2 knockdown significantly suppressed the proliferation of MCF-10A and MDA-MB-231 cells and the transfection of shRNA-resistant full-length *FOXF2* rescued the effect of shFOXF2 on the proliferation of MCF-10A and MDA-MB-231 cells (Figure 1F). Conversely, FOXF2 overexpression in BT549 cells promoted the cell proliferation (Figure 1F). In addition, we also tested the functions of FOXF2 on luminal breast cancer cells by ectopically expressing FOXF2 in MCF-7 cells (see Figure S1A in Additional file 1). However, the exogenous FOXF2 did not significantly affect the migration, invasion and proliferation of MCF-7 cells compared to the control cells (see Figure S1B and C in Additional file 1). Collectively, these results suggest that FOXF2 deficiency enhances the metastatic potential of basal-like mammary epithelial cells and breast cancer cells but reduces cell proliferation.

### FOXF2 deficiency enhances metastatic ability of BLBC cells *in vivo*

**Figure 3:**
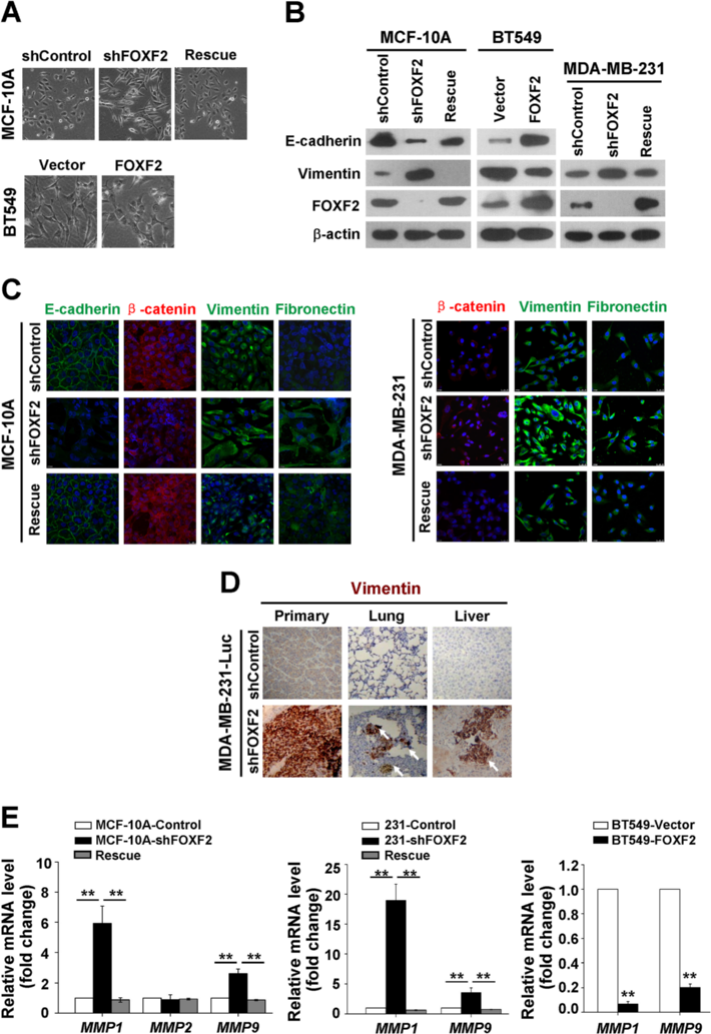
**FOXF2 deficiency induces EMT phenotype of basal-like mammary epithelial cells and BLBC cells. (A)** Morphological photos of the indicated cells (×200). **(B)** The protein expression of EMT markers and FOXF2 in the indicated cells was detected by immunoblot. **(C)** The protein expression of EMT markers in the indicated cells was detected by immunofluorescence staining. **(D)** Vimentin expression in tissues of primary tumor, lung metastases and liver metastases harvested from xenograft mice were stained by immunohistochemistry (×200). Arrows point to the metastatic cancer cells. **(E)** *MMP1*, *MMP2* and *MMP9* mRNA levels in the indicated cells were measured by RT-qPCR. Fold changes were relative to the mRNA expression of the control cells. Three independent assays were performed in triplicate. The data were expressed as mean ± SD. ^**^ *P* <0.01. BLBC, basal-like breast cancer; EMT, epithelial-mesenchymal transition; RT-qPCR, reverse transcription quantitative PCR; SD, standard deviation.

To confirm the role of FOXF2 in metastasis of BLBC cells *in vivo*, 231-Luc-shFOXF2 and 231-Luc-shControl cells were injected into the mammary fat pad of female SCID mice. The general physical condition of mice with 231-Luc-shFOXF2 cells (231-Luc-shFOXF2 mice) was weaker compared to that of mice with 231-Luc-shControl cells (231-Luc-shControl mice) by day 22 after injection (Figure 3A). The weight of 231-Luc-shFOXF2 mice was also lower than that of the 231-Luc-shControl mice (Figure 3B). Consistent with the cell proliferation experiments *in vitro*, 231-Luc-shFOXF2 mice had smaller xenograft tumors than the 231-Luc-shControl mice (Figure 3C).

At day 22 post injection, we performed *in vivo* bioluminescent imaging to monitor the metastasis using a Xenogen IVIS system. The bioluminescent imaging and metastatic photon flux analysis revealed that the 231-Luc-shFOXF2 mice suffered significantly more local invasion and metastatic dissemination than the 231-Luc-shControl mice, especially in the lung and liver metastases (Figure 3D). The data revealed that 80% of 231-Luc-shFOXF2 mice (8/10) suffered metastasis, whereas, 20% of the control mice (2/10) suffered metastasis (Figure 3E). Visible liver metastatic nodules were found in half of the 231-Luc-shFOXF2 mice (5/10), whereas none were found in the livers of the 231-Luc-shControl mice (Figure 3F). H&E staining confirmed that more metastatic foci formed in the lungs and livers in 231-Luc-shFOXF2 mice than in the control mice (Figure 3G). Moreover, there was more muscle invasion in 231-Luc-shFOXF2 mice than in the control mice. The tumors had blood vessel invasion in the xenograft tumors of 231-Luc-shFOXF2 mice but not in the tumors of the control mice (Figure 3H). A Kaplan-Meier survival analysis revealed that the 231-Luc-shFOXF2 mice had a significantly shorter survival time than the 231-Luc-shControl mice (Figure 3I). Taken together, these results indicate that FOXF2 deficiency enhances the metastatic ability of BLBC cells and leads to invasive and metastatic disease *in vivo*.

### FOXF2 deficiency induces EMT phenotype of basal-like mammary epithelial cells and BLBC cells

During mouse embryogenesis, FOXF2 is expressed in the mesenchyme derived from the splanchnic mesoderm adjacent to the ectoderm-derived epithelium and plays an important role in tissue homeostasis through regulating epithelium-mesenchyme interactions to maintain the epithelium polarity [22]. This expression pattern suggests a potential role of FOXF2 in regulating EMT. Therefore, we hypothesized that FOXF2 deficiency in BLBC metastasis might be involved the EMT process. We next investigate whether FOXF2 deficiency induces EMT in BLBC cells. Compared with the MCF-10A-shControl cells, *FOXF2* knockdown transformed MCF-10A cells from epithelial morphology into fibroblast-like shape and caused cell scattering, and the expression of shRNA-resistant full-length *FOXF2* rescued the phenotype (Figure 2A). The detection of EMT markers revealed that, compared to the control cells, *FOXF2*-knockdown cells had reduced level of the epithelial marker E-cadherin in MCF-10A cells (undetectable in the MDA-MB-231 cell lines). Additionally, the *FOXF2*-knockdown cells had elevated levels of the mesenchymal markers vimentin in the MCF-10A, MDA-MB-231 cell lines (Figure 2B). Immunofluorescence staining revealed that the epithelial markers E-cadherin (undetectable in MDA-MB-231) and β-catenin were lost from cell membranes of *FOXF2*-knockdown cells and that the mesenchymal markers vimentin and fibronectin were increased (Figure 2C). The expression of shRNA-resistant full-length *FOXF2* reversed the effect of shFOXF2 on the expression of EMT markers (Figure 2B and C). Immunohistochemistry staining confirmed that vimentin was increased in primary xenograft tumors of 231-Luc-shFOXF2 mice and metastases in lungs and livers (Figure 2D). *FOXF2* knockdown upregulated the expression of *MMP1* and *MMP9* in MCF-10A, MDA-MB-231, and the expression of shRNA-resistant full-length *FOXF2* rescued the effect of shFOXF2 (Figure 2E). Conversely, *FOXF2* overexpression in BT549 cells resulted in a reversal of the EMT phenotype (Figure 2A, B and E). However, the ectopic expression of *FOXF2* in MCF-7 cells did not significantly affect the morphology (see Figure S1D in Additional file 1) and the expression of E-cadherin and vimentin (see Figure S1E in Additional file 1). These observations indicate that FOXF2 functions as an EMT suppressor in basal-like breast cells. FOXF2 deficiency enhances metastatic ability of basal-like mammary epithelial cells and BLBC cells through inducing an EMT phenotype.

### FOXF2 deficiency activates EMT program of basal-like mammary epithelial cells and BLBC cells through upregulation of EMT-TFs

**Figure 4:**
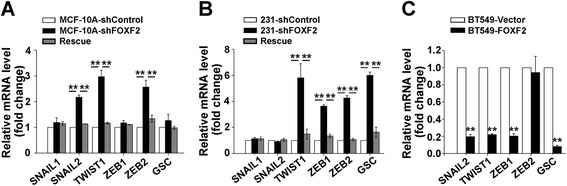
**FOXF2 deficiency activates EMT programming through upregulating EMT-TFs. (A**, **B** and **C)** The mRNA expression levels of EMT-TFs *SNAIL1*, *SNALI2*, *TWIST1*, *ZEB1*, *ZEB2* and *GSC* in the indicated cells were measured by RT-qPCR. Fold changes were relative to the mRNA expression of the control cells. Three independent assays were performed in triplicate. The data were expressed as means ± SD. ^*^ *P* <0.05; ^**^ *P* <0.01. EMT, epithelial-mesenchymal transition; EMT-TFs, EMT-transcription factors; RT-qPCR, reverse transcription quantitative PCR; SD, standard deviation.

To further investigate the mechanisms of how *FOXF2* deficiency induces EMT phenotype, we examined the mRNA expression of EMT-TFs including *SNAIL1*, *SNAIL2*, *TWIST1*, *ZEB1*, *ZEB2* and *GSC* (coding gene of Goosecoid) in MCF-10A, MDA-MB-231 cells with or without *FOXF2* knockdown. The results showed that *FOXF2* knockdown upregulated *TWIST1* and *ZEB2* expression in both MCF-10A and MDA-MB-231 cells (Figure 4A and B). Only *ZEB1* and *GSC* were upregulated in MDA-MB-231 cells and *SNAIL2* was induced in MCF-10A cells (Figure 4A and B). And the expression of shRNA-resistant full-length *FOXF2* rescued the effect of shFOXF2 (Figure 4A and B). Conversely, *FOXF2* overexpression in BT549 cells downregulated *SNAIL2*, *TWIST1*, *ZEB1*, and *GSC* expression (Figure 4C). However, the ectopic expression of *FOXF2* in MCF-7 cells did not significantly change the expression of these EMT-TFs (see Figure S1F in Additional file 1). These results indicate that FOXF2 deficiency induced the EMT phenotype through the activation of different EMT-TFs in the individual basal-like breast cell lines.

### FOXF2 binds to TWIST1 promoter and suppresses its transcription

**Figure 5:**
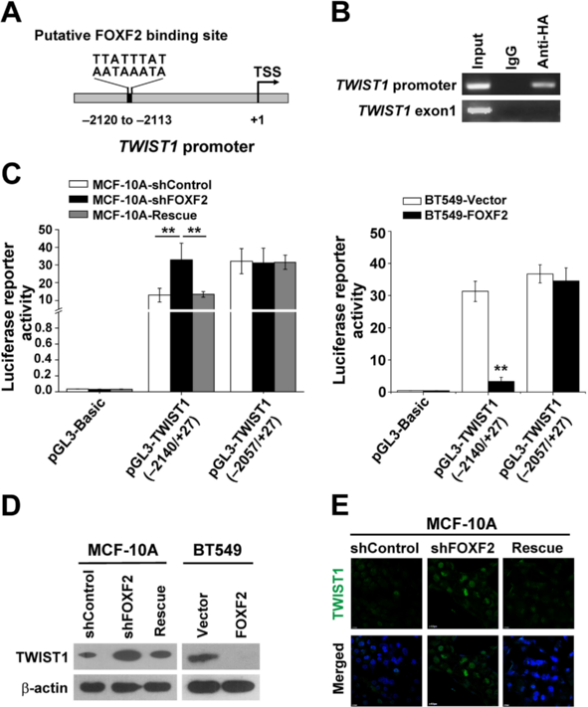
**FOXF2 binds to** ***TWIST1*** **promoter and suppresses** ***TWIST1*** **transcription. (A)** The putative binding site of FOXF2 on the *TWIST1* promoter region. **(B)** The binding of FOXF2 on the *TWIST1* promoter region containing the putative binding site in MCF-10A cells was tested by a ChIP assay. **(C)** Transcriptional activity of *TWIST1* promoter in the indicated cells was assessed by a dual-luciferase reporter assay. **(D)** TWIST1 protein expression in the indicated cells was detected by immunoblot. **(E)** TWIST1 protein expression in the indicated cells was detected by immunofluorescence staining*.* Three independent experiments were performed in triplicate. The data were expressed as means ± SD. ^*^ *P* <0.05; ^**^ *P* <0.01. ChIP, chromatin immunoprecipitation; SD, standard deviation.

Because *TWIST1* mRNA expression levels were commonly and negatively correlated with *FOXF2* expression in the basal-like breast cell lines (Figure 4A, B and C), we investigated whether *TWIST1* is a transcriptional target of FOXF2. First, we performed a BLAST search for FOXF2 binding sites in the promoter region of the *TWIST1* gene. We found a putative FOXF2 binding site AATAAATA in the *TWIST1* promoter region from −2120 to −2113 relative to the transcription start site (TSS; Figure 5A). Subsequently, the binding of FOXF2 on the *TWIST1* promoter region containing or lacking the putative binding site was verified by ChIP assay in MCF-10A cells. The results confirmed that FOXF2 can bind to the *TWIST1* promoter region containing the putative binding site but cannot bind to region lacking the putative binding site (Figure 5B). Therefore, *TWIST1* is a candidate transcriptional target gene of FOXF2.

To further assess the regulatory activity of FOXF2 on the *TWIST1* promoter, we performed a luciferase reporter assay by transfecting pGL3-TWIST1 (−2140 to +27) containing the putative binding site or pGL3-TWIST1 (−2057 to +27) lacking the putative binding site in MCF-10A cells with or without *FOXF2* knockdown. The results showed that the reporter activity of pGL3-TWIST1 (−2140 to +27) was increased in MCF-10A-shFOXF2 cells compared to the control cells, and the expression of shRNA-resistant full-length *FOXF2* rescued the effect of shFOXF2 (Figure 5C). The reporter activity of pGL3-TWIST1 (−2057 to +27) was not significantly changed in *FOXF2*-knockdown cells compared to the control cells (Figure 5C). Conversely, *FOXF2* overexpression in BT549 cells decreased the reporter activity of pGL3-TWIST1 (−2140 to +27; Figure 5C). Furthermore, the role of FOXF2 in negatively regulating TWIST1 protein expression was confirmed using *FOXF2* loss or gain of function in MCF-10A and BT549 cells by immunoblot (Figure 5D) and immunofluorescence detection (Figure 5E). Whereas, the ectopic expression of *FOXF2* in MCF-7 cells did not significantly change the reporter activity of pGL3-TWIST1 (see Figure S1G in Additional file 1) and TWIST1 expression (see Figure S1E in Additional file 1). These results demonstrate that FOXF2 suppress the transcriptional activity of the *TWIST1* promoter in basal-like breast cells. Cumulatively, these data demonstrate that *TWIST1* is a downstream target of FOXF2 and that FOXF2 suppresses *TWIST1* promoter activity and protein expression in basal-like breast cells.

### FOXF2 deficiency induces EMT phenotype of basal-like mammary epithelial cells and aggressive property of BLBC cells via upregulating TWIST1 expression

**Figure 6:**
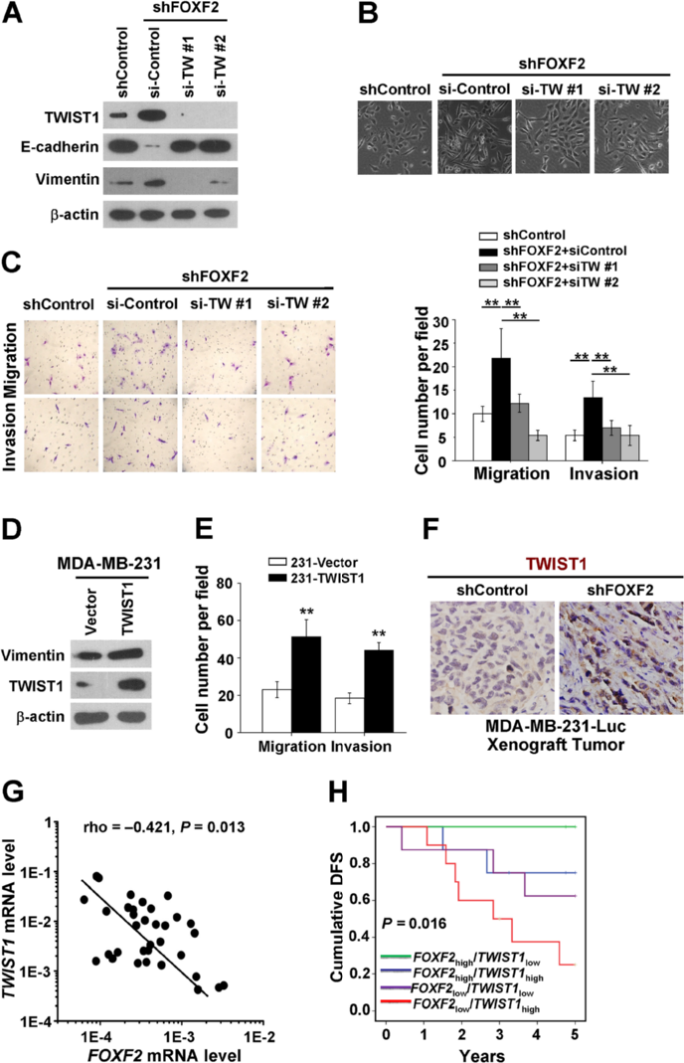
**FOXF2 deficiency induces EMT of basal-like mammary epithelial cells via upregulation** ***TWIST1*** **transcription. (A)** The expression of TWIST1 and EMT markers in the indicated cells was detected by immunoblot. **(B)** Morphology of the indicated cells (×200). **(C)** Photos (Lift) and counts (Right) of migration and invasion of the indicated cells assessed by transwell assay (×200). Three independent experiments were performed in triplicate. The data were expressed as mean ± SD. ^**^ *P* <0.01. **(D)** The expression of TWIST1 and vimentin in the indicated cells was detected by immunoblot. **(E)** The migration and invasion ability of the indicated cells were assessed by transwell assay. Three independent experiments were performed in triplicate. The data were expressed as mean ± SD. ^**^ *P* <0.01. **(F)** The TWIST1 protein expression in primary tumor tissues harvested from xenograft mice of MDA-MB-231-Luc shFOXF2 and MDA-MB-231-Luc shControl cells was stained by immunohistochemistry (×400). **(G)** The correlation of *FOXF2* mRNA levels with *TWIST1* mRNA levels in human primary TNBC tissues (n = 34; Spearman’s correlation rho = −0.421, *P* = 0.013). **(H)** The correlation of combined *FOXF2* and *TWIST1* mRNA levels with the DFS of TNBC patients with *FOXF2*_high_/*TWIST1*_low_ (n = 8), *FOXF2*_high_/*TWIST1*_high_ (n = 8), *FOXF2*_low_/*TWIST*_low_ (n = 8) and *FOXF2*_low_/*TWIST1*_high_ (n = 10) was estimated by a Kaplan-Meier survival analysis. DFS, disease-free survival; EMT, epithelial-mesenchymal transition; SD, standard deviation; TNBC, triple-negative breast cancer.

To further investigate whether TWIST1 mediates the role of FOXF2 deficiency in inducing EMT in basal-like breast cells, we knocked down *TWIST1* expression in MCF-10A-shFOXF2 cells by transiently transfecting two independent *TWIST1* siRNAs (si-TW#1 and si-TW#2), respectively. The results showed that the *FOXF2* knockdown-induced EMT phenotype of MCF-10A cells was reversed by silencing TWIST1 expression. Also, the expression of E-cadherin and vimentin (Figure 6A) was reversed, and epithelial morphology was restored (Figure 6B). Additionally, the cells showed decreased migration and invasion (Figure 6C). Next, we transiently transfected pcDNA3.1-TWIST1 into MDA-MB-231 cells, and the overexpressed TWIST1 in the cells was shown in Figure 6D. We observed that TWIST1 overexpression resulted in elevated levels of the mesenchymal markers vimentin (Figure 6D) and enhanced migration and invasion of the cells (Figure 6E). These results suggested that TWIST may mediate FOXF2-regulated EMT phenotype and aggressive property.

To provide *in vivo* evidence to illustrate the effect of FOXF2 on metastasis was mediated by negatively regulating TWIST1 expression, we detected the TWIST1 protein expression in primary tumors formed by inoculation of MDA-MB-231-Luc shFOXF2 and MDA-MB-231-Luc shControl cells in SCID mice by immunohistochemistry staining. The result showed increased TWIST1 expression in xenograft tumor of MDA-MB-231-Luc shFOXF2 comparing to the control tumor (Figure 6F). Furthermore, we detected the mRNA levels of *FOXF2* and *TWIST1* mRNA in 34 cases of human primary TNBC tissues by RT-qPCR. Consistent with the negative regulation of FOXF2 on the transcription of *TWIST1*, *TWIST1* mRNA levels inversely correlate with the *FOXF2* mRNA levels in TNBC tumor (Spearman’s rho = −0.421, *P* = 0.013; Figure 6G). Then, the TNBC cases were divided into four groups based on *FOXF2* and *TWIST1* mRNA levels: *FOXF2*_high_/*TWIST1*_low_ (n = 8), *FOXF2*_high_/*TWIST1*_high_ (n = 8), *FOXF2*_low_/*TWIST1*_low_ (n = 8) and *FOXF2*_low_/*TWIST1*_high_ (n = 10). Kaplan-Meier analysis showed that the patients in the four subgroups had distinct DFS: the patients in *FOXF2*_high_/*TWIST1*_low_ and *FOXF2*_low_/*TWIST1*_high_ groups had the best and poorest DFS, respectively (*P* = 0.002 between both); the patients in the *FOXF2*_high_/*TWIST1*_high_ and *FOXF2*_low_/*TWIST1*_low_ groups had moderate DFS (*P* = 0.687 between both); *FOXF2*_high_/*TWIST1*_low_ group had better DFS than *FOXF2*_low_/*TWIST1*_low_ (*P* = 0.063) and *FOXF2*_high_/*TWIST1*_high_ (*P* = 0.143) groups; *FOXF2*_low_/*TWIST1*_high_ group had poorer DFS than *FOXF2*_high_/*TWIST1*_high_ (*P* = 0.089) and *FOXF2*_low_/*TWIST1*_low_ (*P* = 0.149) groups (Figure 6H). This result suggests that *TWIST1*_low_ could contribute to the *FOXF2*_high_-suppressed recurrence and metastasis of TNBC cells; conversely, *TWIST1*_high_ could facilitate the *FOXF2*_low_-induced recurrence and metastasis. Taken together, these results demonstrated that TWIST1 functionally mediates the effect of FOXF2 on EMT and metastasis in BLBC cells.

## Discussion

The FOX transcription factors have tissue-specific expression patterns and play critical roles in embryogenesis and tissue development through regulating tissue-specific gene expression and cell differentiation [30]. Recent evidence has demonstrated that the deregulation of FOX factors is correlated with carcinogenesis and tumor progression [21]. It is worth noting that several FOX transcription factors link to the biological characteristics of breast cancer. FOXA1 is highly expressed in luminal subtype breast cancer [31], and its deficiency promotes the metastasis of this subtype cancer [32]. FOXC1 [19], FOXC2 [4], and FOXQ1 [20] are highly expressed in basal-like subtype breast cancer, but are less present in luminal subtype cancer. The ectopic expression of these mesenchymal FOX factors in normal mammary epithelial cells is able to induce EMT and lead to an aggressive phenotype. FOXF2 is a mesenchymal transcription factor and specifically expressed in the mesenchyme adjacent to the epithelium and controls mesenchymal differentiation to maintain the tissue homeostasis via epithelio-mesenchymal interactions [22]. FOXF2 was found highly expressed in benign prostatic hyperplasia and the transition zone of the normal prostate, where less suffer prostate cancer [33,34]. In this study, we showed that FOXF2 is highly expressed in most basal-like breast cells. Since basal-like breast cells are characterized by the basal gene expression and with mesenchymal characteristics [15], the expression pattern of FOXF2 in basal-like breast cells is consistent with its nature of mesenchymal transcription factors. We also found that FOXF2 deficiency promoted the metastasis of BLBC cells, which is consistent with our previous report that the under-expression of FOXF2 is associated with early-onset metastasis and poor prognosis of TNBC patients [23]. Our finding indicates that FOXF2 may play a role in controlling the differentiation state of basal-like breast cells and inhibits the aggressive property of BLBC.

It is well known that a variety of embryonic and mesenchymal transcriptional factors function as EMT activators and are involved in the aggressive behavior of BLBC, including SNAIL1 [16], SNAIL2 [17], TWIST1 [18], and FOX transcription factor family members FOXC1 [19], FOXC2 [4] and FOXQ1 [20]. Most EMT-TFs were identified as activators of EMT and promoters of BLBC metastasis, while EMT suppressors are rarely identified. FOXA2 is one such rare EMT suppressor [35]. In this study, we showed that FOXF2 is a novel EMT-suppressing transcription factor in basal-like breast cells. FOXF2 deficiency enhanced the metastatic ability of basal-like mammary epithelial cells and BLBC cells by inducing an EMT phenotype. We observed that the depletion of *FOXF2* results in E-cadherin and β-catenin loss from the cell membrane. It is known that during EMT progression, the loss of E-cadherin expression from adherent junction results in the release of β-catenin into the cytoplasm. In addition, we observed that the depletion of *FOXF2* leads to a significant increase in *MMPs* expression. Mesenchymal cells and the cancer cells undergoing EMT secrete extracellular matrix (ECM)-degrading enzymes to facilitate invasion and motility [36]. Matrix metalloproteinase (MMP)2 [37], MMP3 [38] and MMP9 [39] are able to induce EMT, and they trigger a positive regulatory feedback loop that stabilizes EMT. Therefore, the translocation of β-catenin translocation and upregulation of MMPs caused by FOXF2 deficiency could lead to initiate vicious cycle of EMT.

TWIST1 is recognized as a critical modulator in EMT, and its overexpression is able to trigger EMT phenotype and tumor metastasis [3]. Thus, clarifying the regulatory mechanism of TWIST1 expression would provide insight into the regulatory pathway of TWIST1-induced EMT programming and aid in developing molecular targets to inhibit tumor metastasis. It has been reported that HIF-1 [40] and p65 [41] transcriptionally activate *TWIST1* expression to mediate hypoxia-induced and tumor necrosis factor alpha (TNF-α)-induced EMT and tumor metastasis, respectively. miR-720 [42] and miR-106b [43] suppress TWIST1 expression by posttranscriptional regulatory mechanisms. In this study, we identified FOXF2 as a transcriptional suppressor of TWIST1. Therefore, we speculate that the two embryonic/mesenchymal transcription factors might act cooperatively to maintain tissue homeostasis through balancing the differentiation or dedifferentiation of mesenchymal/myoepithelial cells. FOXF2 deficiency could lead to more dedifferentiation of myoepithelial cells and an aggressive phenotype in BLBC via a TWIST1-mediated pathway. This novel finding provided an unanticipated regulatory pathway of TWIST-induced EMT and metastasis in BLBC.

In EMT programming, pleiotropic EMT-TFs form an interaction network to enable them to work in concert to regulate the EMT phenotype. The zinc-finger transcription factors SNAIL1 [44], SNAIL2 [45], ZEB1 [46] and ZEB2 [47] directly bind to the E-boxes of the *CDH1* promoter to repress its transcription. TWIST1 [3], Goosecoid [48], and FOXC2 [4] trigger EMT by indirectly suppressing *CDH1* transcription. TWIST1 directly regulates *SNAIL2* transcription, and SNAIL2 is an essential mediator of TWIST1-induced EMT [49]. TWIST1 can also induce FOXC2 and SNAIL2 expression [7]. SNAIL1 has been found to activate ZEB1 [2], FOXC2 and SNAIL2 expression [7]. In this study, we found that FOXF2 not only negatively regulated *TWIST1* expression but also regulated the expression of *SNAIL1* and *SNAIL2*, *ZEB1*, *ZEB2* and *GSC* in basal-like cell lines. Interestingly, FOXF2 regulated *TWIST1* expression in all the three cell lines, but only regulated *SNAIL2* in MCF-10A and BT-549 cells, regulated *ZEB1* and *GSC* in MDA-MB-231 and BT-549 cells, regulated *ZEB2* in MCF-10A and MDA-MB-231 cells. Therefore, in addition to activating *TWIST1* transcription, FOXF2 deficiency may induce EMT through orchestrating different EMT-TFs expression in the different basal-like breast cells.

In addition to BLBC, we also tested the effect of FOXF2 on luminal breast cancer cells. However, we observed that the ectopic expression of FOXF2 could not significantly change the phenotype of luminal breast cancer cells, even slightly enhance the migration and invasion, and slightly increase the transcription of *TWIST1*. Our results suggest that there are different functions of FOXF2 in basal-like and luminal cells. Based on our current results, we speculate that mesenchymal FOXF2 might be induced expression in luminal cells that had undergone transdifferentiation into basal-like cells, and when FOXF2 depletion during the progression of the cells adapted mesenchymal characteristics could drive these cells to possess more mesenchymal stem-like plasticity and aggressive phenotype by activating the transcription of target gene *TWIST1* and upregulating the expression of other EMT-TFs. The regulatory mechanisms of the different expression pattern and functions of FOXF2 in basal-like and luminal breast cancer cells should be further deeply investigated. Since FOXF2 plays an essential role in the metastasis of BLBC cells, the exogenous administration of FOXF2 or interference of FOXF2-TWIST1 pathway may be a promising therapeutic strategy for improving the outcome of BLBC patients.

## Conclusions

In conclusion, our study identified FOXF2 as a novel EMT-suppressing transcription factor in BLBC. FOXF2 deficiency enhances metastatic ability of BLBC cells via activating an EMT program by upregulating the transcription of *TWIST1*.

## Additional file

Additional file 1: Figure S1.
*FOXF2* overexpression was incapable of inducing EMT of luminal breast cancer cells*.* **(A)** The protein expression of FOXF2 in the indicated cells was detected by immunoblot. **(B)** The migration and invasion ability of the indicated cells were assessed by transwell assay. **(C)** The proliferation ability of the indicated cells was determined by MTT assay. **(D)** Morphological photos of the indicated cells (×200). **(E)** The protein expression of EMT markers in the indicated cells was detected by immunoblot. **(F)** The mRNA expression levels of EMT-TFs *SNAIL1*, *SNALI2*, *TWIST1* and *GSC* in the indicated cells were measured by RT-qPCR. Fold changes were relative to the mRNA expression of the control cells. **(G)** Transcriptional activity of *TWIST1* promoter in the indicated cells was assessed by a dual-luciferase reporter assay. Three independent assays were performed in triplicate. The data were expressed as mean ± SD. *n.s*., *P* >0.05.

## Abbreviations

bHLH: basic helix-loop-helix
BLBC: basal-like breast cancer
ChIP: chromatin immunoprecipitation
CK: cytokeratins
DFS: disease-free survival
ECM: extracellular matrix
EGFR: epithelial growth factor receptor
EMT: epithelial-mesenchymal transition
EMT-TFs: EMT-transcription factors
ER: estrogen receptor
FBS: fetal bovine serum
FOX: forkhead box
GAPDH: glyceraldehyde 3-phosphate dehydrogenase
GOBO: Gene expression-based Outcome for Breast cancer Online
H&E: hematoxylin and eosin
HER2: human epidermal growth factor receptor 2
MMP: matrix metalloproteinase
MTT: 3-(4,5-dimethylthiazol-2-yl)-2,5-diphenyltetrazolium bromide
PBS: phosphate-buffered saline
PR: progesterone receptor
RT-qPCR: reverse transcription quantitative PCR
SCID: severe combined immunodeficiency
SD: standard deviation
shRNA: short hairpin RNA
siRNA: small interfering RNA
TMUCIH: Tianjin Medical University Cancer Institute and Hospital
TNBC: triple-negative breast cancer
TNF-α: tumor necrosis factor alpha
TSS: transcription start site
