# Supplementary figures and images for: FOXF2 deficiency promotes epithelial-mesenchymal transition and metastasis of basal-like breast cancer
Source: Breast Cancer Res. 2015 Feb 26;17(1):30. doi: 10.1186/s13058-015-0531-1 (PMC4361145; doi:10.1186/s13058-015-0531-1)

# Figure S1

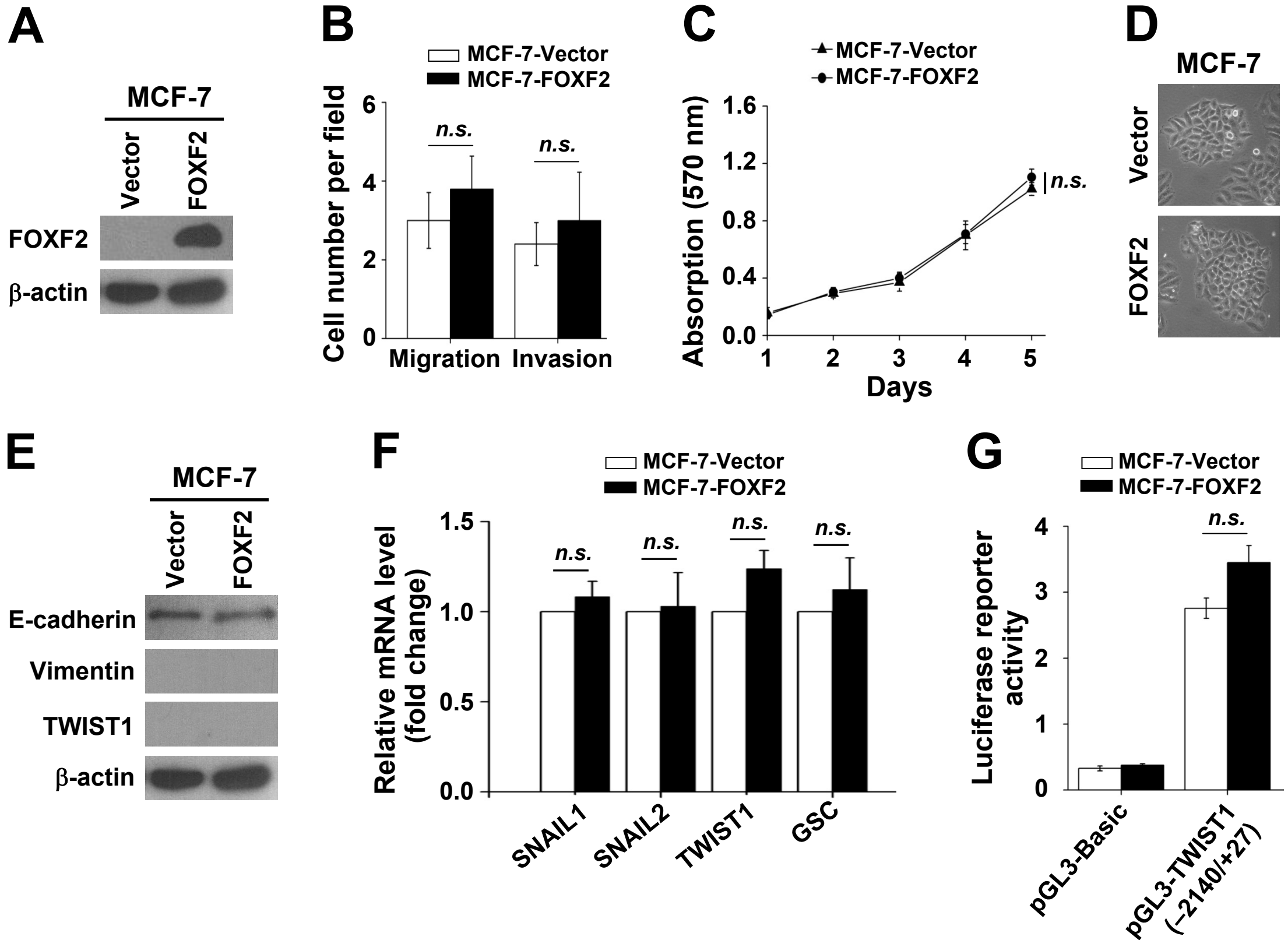

Supplement: Additional file 1: Figure S1. — FOXF2 overexpression was incapable of inducing EMT of luminal breast cancer cells. (A) The protein expression of FOXF2 in the indicated cells was detected by immunoblot. (B) The migration and invasion ability of the indicated cells were assessed by transwell assay. (C) The proliferation ability of the indicated cells was determined by MTT assay. (D) Morphological photos of the indicated cells (×200). (E) The protein expression of EMT markers in the indicated cells was detected by immunoblot. (F) The mRNA expression levels of EMT-TFs SNAIL1, SNALI2, TWIST1 and GSC in the indicated cells were measured by RT-qPCR. Fold changes were relative to the mRNA expression of the control cells. (G) Transcriptional activity of TWIST1 promoter in the indicated cells was assessed by a dual-luciferase reporter assay. Three independent assays were performed in triplicate. The data were expressed as mean ± SD. n.s., P >0.05. [file 13058_2015_531_MOESM1_ESM.pdf]
